# First report of whole-genome analysis of an extensively drug-resistant *Mycobacterium tuberculosis* clinical isolate with bedaquiline, linezolid and clofazimine resistance from Uganda

**DOI:** 10.1186/s13756-022-01101-2

**Published:** 2022-05-12

**Authors:** Jupiter Marina Kabahita, Joel Kabugo, Francis Kakooza, Isa Adam, Ocung Guido, Henry Byabajungu, Joanitah Namutebi, Maria Magdalene Namaganda, Pius Lutaaya, James Otim, Fredrick Elishama Kakembo, Stephen Kanyerezi, Patricia Nabisubi, Ivan Sserwadda, George William Kasule, Hasfah Nakato, Kenneth Musisi, Denis Oola, Moses L. Joloba, Gerald Mboowa

**Affiliations:** 1 National Tuberculosis Reference Laboratory/Supranational Reference Laboratory, Plot 106-1062 Butabika Road, Luzira, Uganda; 2 Africa Centres for Disease Control and Prevention, African Union Commission, Roosevelt Street, P.O. Box 3243, W21 K19 Addis Ababa, Ethiopia; 3 The Infectious Diseases Institute, College of Heath Sciences, Makerere University, P.O. Box 22418, Kampala, Uganda; 4 Department of Immunology and Molecular Biology, College of Health Sciences, Makerere University, P.O. Box 77072, Kampala, Uganda; 5 The African Center of Excellence in Bioinformatics and Data-Intensive Sciences, The Infectious Diseases Institute, Makerere University, P.O Box 22418, Kampala, Uganda; 6 Lira Regional Referral Hospital, P.O. Box 2, Lira, Uganda

**Keywords:** Bedaquiline, Linezolid, Clofazimine, Extensively drug-resistant TB (XDR-TB), Whole-genome sequencing (WGS), *Mycobacterium tuberculosis*

## Abstract

**Background:**

Uganda remains one of the countries with the highest burden of TB/HIV. Drug-resistant TB remains a substantial challenge to TB control globally and requires new strategic effective control approaches. Drug resistance usually develops due to inadequate management of TB patients including improper treatment regimens and failure to complete the treatment course which may be due to an unstable supply or a lack of access to treatment, as well as patient noncompliance.

**Methods:**

Two sputa samples were collected from Xpert MTB/RIF® assay-diagnosed multi-drug resistant tuberculosis (MDR-TB) patient at Lira regional referral hospital in northern Uganda between 2020 and 2021 for comprehensive routine mycobacterial species identification and drug susceptibility testing using culture-based methods. Detection of drug resistance-conferring genes was subsequently performed using whole-genome sequencing with Illumina MiSeq platform at the TB Supranational Reference Laboratory in Uganda.

**Results:**

In both isolates, extensively drug-resistant TB (XDR-TB) was identified including resistance to Isoniazid (*katG* p.Ser315Thr), Rifampicin (*rpoB* p.Ser450Leu), Moxifloxacin (*gyrA* p.Asp94Gly), Bedaquiline (*Rv0678* Glu49fs), Clofazimine (*Rv0678* Glu49fs), Linezolid (*rplC* Cys154Arg), and Ethionamide (*ethA* c.477del). Further analysis of these two high quality genomes revealed that this 32 years-old patient was infected with the Latin American Mediterranean TB strain (LAM).

**Conclusions:**

This is the first identification of extensively drug-resistant *Mycobacterium tuberculosis* clinical isolates with bedaquiline, linezolid and clofazimine resistance from Uganda. These acquired resistances were because of non-adherence as seen in the patient’s clinical history. Our study also strongly highlights the importance of combating DR-TB in Africa through implementing next generation sequencing that can test resistance to all drugs while providing a faster turnaround time. This can facilitate timely clinical decisions in managing MDR-TB patients with non-adherence or lost to follow-up.

**Supplementary Information:**

The online version contains supplementary material available at 10.1186/s13756-022-01101-2.

## Background

Uganda is a high burden country for both HIV and Tuberculosis (TB) [1]. Worldwide, drug resistant forms of TB undermine the efforts to curb TB disease. An estimated 1500 people fell ill with drug resistant TB (DR-TB) in 2018 with only 34% of these being notified to the Uganda National TB Program with treatment success rate for DR-TB of only 64% [2]. Between 2014 and 2018, the incidence of rifampicin-resistant tuberculosis (RR-TB) increased especially among newly diagnosed TB patients with males being more affected by RR-TB than females [3]. A number of challenges such as patient adherence, long duration of treatment and late diagnosis have also been linked to treatment failure in TB therapy [4]. Particularly, longer regimens involved in the treatment of DR-TB can potentially explain the higher non-adherence and treatment defaulting seen in these patients [5].

Multi-Drug Resistant TB (MDR-TB) is TB that is resistant to atleast Rifampicin and Isoniazid. These are the two most effective anti-TB drugs and resistance to them warrants the use of second line anti-TB drugs that are less effective, more costly, and more toxic [6]. Previously, extensively drug resistant TB (XDR-TB) was defined as MDR-TB that had additional resistance to any fluoroquinolones and any one of the second line injectables (Amikacin, Capreomycin and Kanamycin). This definition has now been redefined to reflect changes in policy and treatment guidelines as there has been a declining priority in use of the second line injectables and increased importance of drugs like Bedaquiline and Linezolid. XDR-TB currently, is defined as MDR-TB that is resistant to a fluoroquinolone and either Bedaquiline or Linezolid or both [6].

Bedaquiline, Linezolid and Clofazimine have over the years gained importance in treatment of drug resistant TB, Bedaquiline and Linezolid are now classified as Group A second line drugs while Clofazimine is classified as a Group B [6]. Resistance to these drugs is still rare but has been reported elsewhere [7–9] including TB drug naive patients [10–12]. Therefore, monitoring resistance to these drugs is of paramount importance given they are now a part of important regimens to treat resistant TB.

Cross resistance to Bedaquiline and Clofazimine has been attributed to mutations occurring in genes *Rv0678*, *mmpS5-mmpL5*, *Rv1979c*, and *pep*Q. In addition to these genes, mutations in the *atpE* gene which is the primary target for Bedaquiline cause a much higher level of resistance to Bedaquiline, these mutations are not known to confer cross-resistance to Clofazimine [9, 10, 13, 14]. Resistance to Linezolid has been attributed to mutations occurring in genes *rplC* and *rrl* [7].

In this report, we present the first identified XDR clinical isolate of *Mycobacterium tuberculosis* (M. tb) that had confirmed phenotypic resistance to Bedaquiline, Linezolid and Clofazimine. This resistance was displayed by two sputa samples from the same patient collected at different time points. To identify the possible genetic causes of resistance in these samples, Whole-genome sequencing (WGS) of both isolates was carried out.

## Case presentation

A 32-year-old male patient with HIV negative sero-status was admitted at Lira Regional referral hospital in Northern Uganda as a failure on the first line regimen (Rifampicin, Isoniazid, Ethambutol and Pyrazinamide). At admission, the patient had a baseline weight of 50 kg and Chest X-ray conducted showed heterogeneous opacities bilaterally, with obliterated costophrenic angles bilaterally. The patient was confirmed Rifampicin Resistant (RR) by Xpert MTB/RIF assay on 30/7/2017 from a lower peripheral facility with cycle threshold (ct) values of 10. A sputum sample was first collected from the patient and referred to the National Tuberculosis Reference Lab (NTRL) for culture and drug susceptibility testing. The patient was initiated on DR-TB treatment regimen composed of Kanamycin (800 cc), Levofloxacin(1000 mg), Ethionamide (750 mg), Cycloserine (500 mg), and Pyrazinamide (1200 mg) on 31/7/2017. Results from the patient’s initial sample were 3+ for acid-fast bacillus (AFB) smear microscopy*, M. tb* Colony growth of 3+ on Lowenstein Jensen media (LJ) and confirmed positive for *M. tb* on Mycobacteria Growth Indicator Tube (MGIT) liquid culture system with a time to detection (TTD) of 4 days and 12 h. Routine phenotypic drug susceptibility (p-DST) testing results for this baseline sample collected on 31/08/2017 confirmed resistance to both Rifampicin and Isoniazid and susceptibility to Fluroquinolones, Ethambutol and the second-line injectables. Month one follow-up results were negative for acid fast bacilli on smear microscopy but had *M. tb* colony growth of 1+ on LJ and positive for *M. tb* on MGIT culture with a TTD of 19 days and 4 h. Follow-up at month two had negative results for both smear microscopy and mycobacterial culture. Patient was declared lost to follow-up (LTFU) on 12/12/2017.

The patient was re-registered on 12/05/2018 and initiated on the same DR-TB treatment regimen *6 km Lfx Cs Eto Z /14 Lfx Cs Eto Z*. Results from the sample referred to the NTRL at re-registration were 3+ for AFB smear microscopy, colony growth of 3+ on LJ culture media and confirmed positive on MGIT culture with a TTD of 5 days and 7 h. The patient’s mycobacterial culture converted at month four on 08/09/2018 but later reverted at month six (07/11/2018). The results for month six reversion showed negative AFB smear microscopy, colony growth of 2+ on LJ, confirmed positive MGIT culture with a TTD of 10 days and 11 h as well as resistance to fluoroquinolones both p-DST and Line probe assay. p-DST also revealed resistance to Streptomycin at this point. p-DST done on 08/05/2019 for Clofazimine and Bedaquiline for follow-up at month twelve showed susceptibility to both drugs but still resistant to Streptomycin, fluoroquinolones, Rifampicin, and Isoniazid. The patient was declared a treatment failure on 03/06/2019.

The patient was re-registered on 29/07/2019 on a regimen containing 24 Bdq Dlm Lzd Eto Cs Z as recommended by the Ugandan National MDR panel, the culture converted at month 5 but reverted at month 6, p-DST for follow-up month 7 still showed sensitivity to Bedaquiline, Clofazimine and Linezolid. The patient was LTFU after month 10 since his TB treatment got interrupted for more than two consecutive months. He was later re-registered on 2/6/2020 on the regimen 12 Bdq Lzd Dlm Cfz Cs 6 Lzd Cfz Cs. Follow-up p-DST at month two (M2) and three (M3) on this new regimen showed resistance to Bedaquiline, Clofazimine and Linezolid. The patient was declared LTFU 02/02/2021 and his whereabouts remain unknown to date.

## Materials and methods

### Phenotypic drug susceptibility testing and isolate identification

All laboratory processes in this study were carried out at the NTRL-Uganda/World Health Organization (WHO) Supra-National Tuberculosis Reference laboratory (SRL). Routinely all MDR-TB patients in Uganda are followed-up every month for 24 months with samples being collected at their respective treatment centers and referred to NTRL-Uganda for comprehensive laboratory analysis. In this case report, we describe two patient samples (M2 and M3) collected at Lira regional referral hospital as part of TB patient care. Each sample received was processed using 6.0% sodium hydroxide (NaOH) in the N-acetyl-L-cysteine-sodium hydroxide (NALC-NaOH) mixture before performing Auramine-O smear microscopy, mycobacterial culture, and drug susceptibility on both MGIT and LJ methods (Additional file 1 and 2 respectively), all these are carried out as part of TB patient care. On both LJ and the BACTEC MGIT 960 system, samples were tested for resistance to both first and second anti-TB drugs using WHO-recommended critical concentrations [15] and NTRL-Uganda in-house protocols as described in Additional file 1 and 2. Drugs that were tested included Isoniazid, Ethambutol and Rifampicin at critical concentrations. Second-line anti-TB drugs Levofloxacin, Moxifloxacin and Amikacin were tested on both MGIT and LJ methods. In addition to MGIT drugs, we also tested other drugs such as Linezolid, Bedaquiline, Clofazimine and Delamanid. Quality control using *M. tb* strains H37RV, MX and KA was done to ensure reliability of results obtained. Control strains H37Rv catered for sensitivity of both first line and second line drugs whereas MX and KA catered for sensitivity and resistance to both first- and second-line drugs. The two samples described here were chosen for WGS based on their phenotypic drug susceptibility test results that showed the first instance of Bedaquiline, Clofazimine and Linezolid resistance recorded in clinical isolates (Additional file 3).

### Sub-culture and DNA extraction

The isolates were sub-cultured on selective Middlebrook 7H11 agar (Becton and Dickson, USA) and incubated at 37 °C in a CO_2_ incubator (Panasonic, Osaka, Japan) monitored weekly for mycobacterial growth. During the 4th week, sufficient colonies were observed, and these were harvested into a 15 ml falcon tube with 1 ml of sterile water. Following a 30 min heat inactivation at 85 °C, high quality genomic DNA was extracted using an in-house cetyl trimethylammonium bromide (CTAB) method as previously described [16]. Quality of the extracted DNA was assessed using the 4150 TapeStation system (Agilent, USA) with the Agilent Genomic DNA Screen Tape and reagents. Purity of the DNA was assessed using the Nano-Drop 2000c (Thermo-Fisher Scientific).

### Library preparation and whole-genome sequencing

Genomic libraries were prepared using the Illumina Nextera XT library preparation kit following manufacturer’s instruction [17]. Quality of the prepared genomic libraries was assessed with the Agilent 4150 TapeStation system using the D1000 High sensitivity Screen Tape and reagents. Genomic libraries were sequenced on the MiSeq platform (Illumina, San Diego, CA, USA) using the Illumina MiSeq V3 cartridge.

### Bioinformatics analysis

#### Drug resistance determination and variant calling

Quality of reads was assessed using FastQC v0.11.8 [18] and MultiQC v1.0 [19]. TB-Profiler v3.0.4 [20] pipeline was run to identify resistance to the different anti-TB drugs. For potential novel mutations conferring resistance to the newly approved anti-TB drugs, the pipeline described next was used. Genomic reads were aligned to the *M. tb* H37Rv NC_000962.3 reference genome using BWA v0.7.17-r1188 [21] with the mem algorithm generating SAM files which were converted into BAM files using SAMtools v1.9 [22]. These SAM files were subsequently sorted and indexed using the same version of SAMtools. Variant calling was done using FreeBayes v1.3.2 [23], a variant was only called if the read depth at that position was greater than 10 and the variant had a Phred quality score of 15 or higher.

Variants were annotated using SnpEff v4.3 [24]. Custom bash scripts were used to identify potential drug resistance-conferring mutations in the genes *Rv0678*, *mmpL5*, *mmpS5*, *pepQ*, and *Rv1979c* for both Bedaquiline and Clofazimine resistance. In addition to these genes, the *atpE* gene was included for Bedaquline resistance. Other genes including *fbiA*, *fbiB*, *fbiC*, *Rv2983*, *fgd1*, and *ddn* were interrogated for potential resistance-conferring gene variants to Delamanid and Pretomanid while for Linezolid resistance, the genes *rplC* and *rrl* were interrogated.

### Phylogenetic analysis

**Fig. 1:**
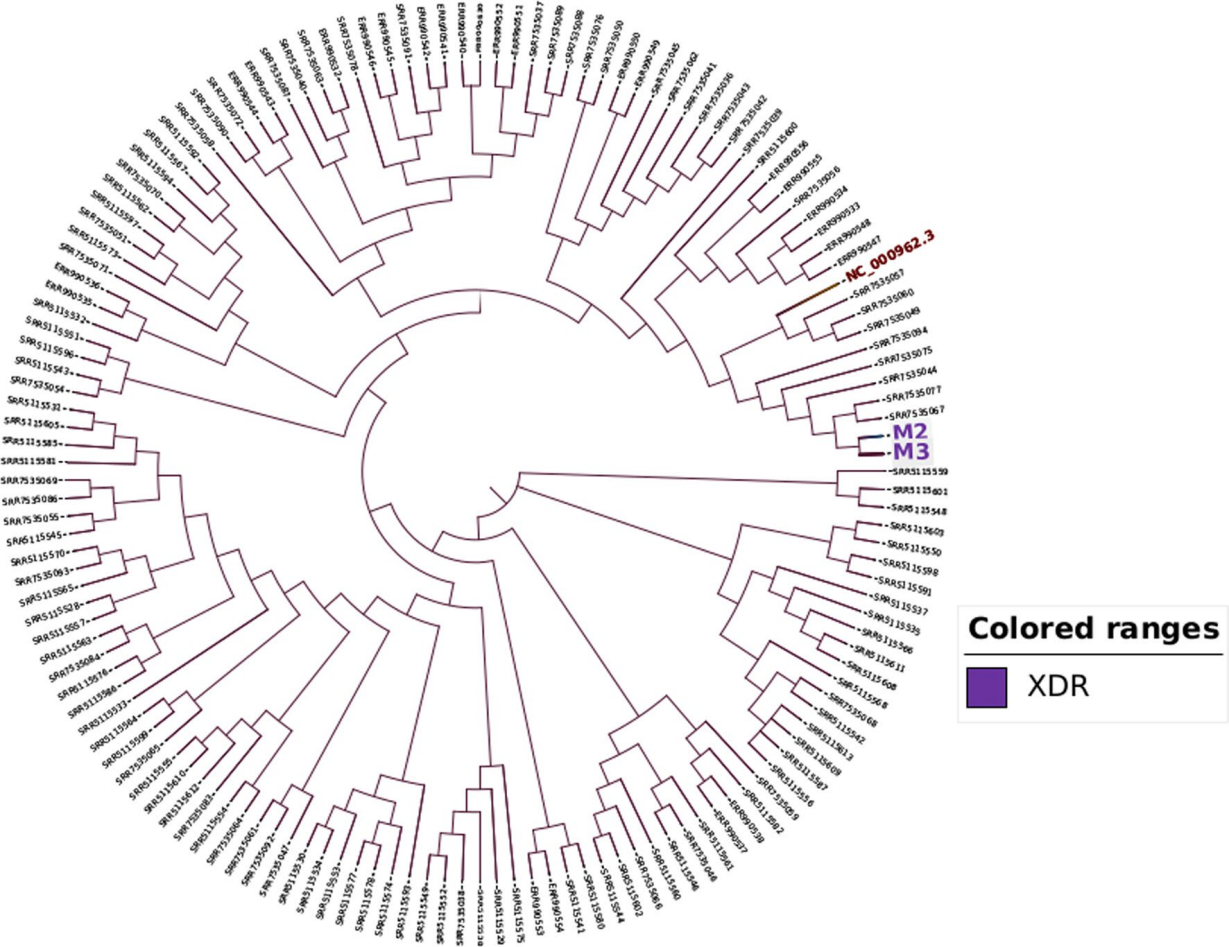
Phylogenetic relationship between the two XDR samples and other 169 sequences from Uganda

Phylogenetic analysis of the samples was done alongside 169 M*. tb* samples from Uganda under the Sequence Read Archive experiment numbers PRJEB10577, PRJNA354716 and PRJNA481638 (Fig. 1). The samples were called for variants using snippy v4.6.0 [25]. Twenty-four of the samples were excluded from the downstream analysis as these flagged a “[markdup] error.

To build a high-resolution phylogeny, a snp core alignment was first performed using snippy core [25]. Gubbins v2.4.1 [26] was used to produce an alignment file with recombinant sites masked. Snp-sites v2.5.1 [27] was then used to clean the alignment and only retain sites with exclusively ACGT. Using Fasttree v2.1.10 [28], a tree for a nucleotide alignment with the GTR + CAT substitution model was inferred. To visualize the tree, iTOL v.6.3 [29] was used to display and manipulate the phylogenetic tree.

## Results

### Resistance associated mutations to the different anti-TB drugs

Our analysis showed that there was agreement between MGIT and WGS drug resistance results. Analysis of both sequences using TB-Profiler confirmed resistance conferring mutations *rpoB* p.Ser450Leu, *katG* p.Ser315Thr, *gyrA* p.Asp94Gly, *rplC* p.Cys154Arg known to cause resistance to drugs Rifampicin, Isoniazid, fluoroquinolones, and Linezolid respectively (Table 1).

**Table 1:** Mutations identified conferring resistance to the different anti-TB drugs

Drug	Resistance profile	Gene mutation
Rifampicin	Resistant	*rpoB* p.Ser450Leu
Isoniazid	Resistant	*katG* p.Ser315Thr
Fluroquinolones	Resistant	*gyrA* p.Asp94Gly
Ethionamide	Resistant	*ethA* c.477del
Bedaquiline	Resistant	*Rv0678* p.Glu49fs
Clofazimine	Resistant	*Rv0678* p.Glu49fs
Linezolid	Resistant	*rplC* p.Cys154Arg

### Other identified mutations in the genes conferring resistance to the bedaquiline, clofazimine, linezolid, and delamanid

TB-Profiler was only able to confirm genotypic resistance to Linezolid and other drugs, but because the isolates were displaying phenotypic resistance to Bedaquiline and Clofazimine, we decided to interrogate the genes that have so far been implicated in resistance to both drugs for potential novel resistance conferring mutations. Our analysis identified several mutations in *mmpl5*, *pepQ*, *Rv0678*, and *Rv1979c* (Table 2). *Rv0678* frame shift p.Glu49fs was confirmed to confer resistance to Bedaquiline and cross-resistance to Clofazimine [14], its occurrence in our two samples could potentially explain the observed phenotypic resistance to both drugs. No mutations were seen in the *atpE* gene for both samples.

**Table 2:** Other potential resistance conferring mutations in the genes implicated in Bedaquiline, Clofazimine, Linezolid and Delamanid resistance

Drug	Gene	Other potential resistance conferring mutations	Mutation type
Bedaquilin and Clofazimine	*mmpL5*	p.Ile948Val, p.Gly131Arg, p.Thr352Pro	Missense variants
	*Rv0678*	p.Ala124Gly, p.Asp141Glu, p.Thr161Asn,	Missense variants
	*Rv1979c*	p.Ala128Ser, p.Pro349Ala	Missense variants
	*pepQ*	p.Ala224Ser, p.Leu300Arg, p.Leu362Trp,	Missense variants
Linezolid	*rplC*	p.Gly153Glu p.Ala155Asp, p.Thr156Pro	Missense variant
Delamanid	*fgd1*	p.Ser38Arg, p.Thr92Pro, p.Phe102Cys, p.Gly199Ala, p.Glu213Asp	Missense variants
	*fbiC*	p.Cys59Gly, p.Asp375Ala, p.Glu376Gly, p.Trp405Gly, p.Ile406Ser, p.Asp492Tyr, p.Glu503Gly, p.Ile638Ser, p.Leu672Val,p.Leu672Arg, p.Asp673Ala, p.Pro686Ala, p.Glu692Ala,	Missense variants
		p.Glu152*	Nonsense mutation
	*fbiA*	p.Val73Gly, p.Met319Arg	Missense variants
	*fbiB*	p.Ile18Val, p.Ser54Ala, p.Ala104Glu, p.Leu249Arg, p.Glu250Ala, p.Ser262Ala, p.Val263Gly,	Missense variants
	*ddn*	p.Glu35Lys, p.Glu35Gly, p.Asp108Glu	Missense variants
	*Rv2983*	p.His159Pro	Missense variant

The role of other mutations (Table 2) seen in these gene remains to be confirmed, only the mutation *mmpL5* p.Ile948Val has been confirmed not to be linked to drug resistance [1, 30]. We identified several mutations in genes implicated in resistance to Delamanid: *fbiA*, *fbiB*, *fbiC*, *Rv2983*, *fgd1*, as well as *ddn*. *fbiC* carried more mutations than other genes. Some of the mutations observed in one of the samples were at the same genomic position [(fbiC p.Leu672Val and p.Leu672Arg), (ddn p.Glu35Lys, p.Glu35Gly)] which might be explained by the heterogeneous nature of the samples.

### Lineage analysis

Lineage results from TB-Profiler showed that both samples belonged to MTBC lineage 4 sub-lineage LAM.

### Analysis of variants

From the variant analysis, 696 variants were shared between M3 and M2. Eighty-four variants were exclusive to M3 and not M2. One variant only is present in M2 and not in M3. Isolate M3 accumulated more variants including complex variants (COMPLEX), deletions (DEL), insertions (INS), Multiple Nucleotide Polymorphism (MNP), single nucleotide polymorphisms (SNP), compared to M2 on average (Table 3).

**Table 3:** Variants distribution in the two DR-TB M. tb clinical isolates (M2 was the first sample to be collected from the patient)

Type	M2	M3
Variant-complex	13	15
Variant-DEL	29	36
Variant-INS	25	36
Variant-MNP	3	5
Variant-SNP	627	688
Total variants	697	780

## Discussion

Global control of tuberculosis continues to be impacted by the emergence and transmission of DR-TB which is associated with increased morbidity and mortality due to reduced treatment success. Current microbiological culture-based and molecular diagnostic tools such as Xpert MTB/Ultra and Line Probe Assay have limitations that could lead to delay of appropriate treatment initiation causing transmission of drug resistance. Furthermore, detection of DR-TB in Uganda relies on Xpert MTB/RIF and line probe assays (MTBDRplus and MTBDRsl). These assays are limited to detecting resistance to only first-line and second-line drugs but not to new anti-TB drugs in the treatment of MDR-TB. The Supranational TB Reference Laboratories (SRLs) should adopt next-generation sequencing (NGS) and easy to use bioinformatics pipelines for comprehensive rapid detection, monitoring and surveillance of *M. tb* drug resistance as well as identify their novel mechanisms of action.

One person with active untreated TB can spread the disease to as many as 15 other people in a year [31]. Our case was staying with his mother who also got infected with DR-TB however she was successfully treated and discharged in July 2021. About 15 percent of TB patients in Uganda abandon treatment and remain in the community settings [32]. The patient in this study exhibited very poor adherence to treatment behavior. He was declared LTFU twice on 12/12/2017 and 2/02/2021. This underscores the need for adequate clinical monitoring of these patients through modified directly observed treatment strategy (DOTS-Plus) which requires much effort from both the patients and health-care workers during treatment.

The quinolone resistance-determining region (QRDR) is comprised of conserved areas within *gyrA* and *gyrB*. Isolates harboring the *gyrA* Asp94Gly substitution have been reported to resist higher fluoroquinolones concentrations [33]. Similarly, mutations within the *ethA* structural genes are associated with relatively high levels of ethionamide (ETH) resistance [34]. The isolates in this report carried *ethA* c.477del.

Clofazimine has been repurposed for the treatment for MDR-TB [35]. But mutations in Rv0678 are a major mechanism of clofazimine-bedaquiline cross-resistance [36]. Rv0678 p.Glu49fs mutation identified in these isolates has also been reported by a study in KwaZulu-Natal, South Africa [14].

Another recent study has showed that 6.3% of MDR-TB patients without prior clofazimine or bedaquiline exposure harbored isolates with Rv0678 mutations, which raises concern that pre-existing resistance to these drugs may be associated with prior TB treatment [35]. The study recommended extensive surveillance and personalized testing for clofazimine and bedaquiline resistance, together with assessment of their clinical usage, especially among previously treated and MDR-TB patients [35]. The *rplC* p.Cys154Arg also identified in this study has been identified as a dominant mutation in linezolid-resistant *M. tb* strains elsewhere [37]. The patient in this study showed resistance to almost all new anti-TB drugs (bedaquiline, linezolid and clofazimine) that are currently suggested as priority drugs for MDR-TB treatment. Therefore, this study underscores the need to support TB treatment adherence. This study did not find any phenotypic or genomic pyrazinamide resistance. According to Uganda Programmatic Management of drug-resistant Tuberculosis (PMDT) guidelines, all newly diagnosed RR-TB/MDR-TB patients are started on a standard regimen—6 km-Lfx-Cs-Eto-Z/14Lfx-Cs-Eto-Z until their DSTs are available at which point, their treatment becomes individualized based on observed resistance and side effects profiles [38].

## Conclusion and recommendation

We have documented a first report describing identification of extensively drug-resistant *M. tb* clinical isolates with Bedaquiline, Linezolid and Clofazimine resistance from Uganda through Whole-genome sequence analysis. These acquired resistances were because of non-adherence as seen in the patient’s clinical history. This finding has important implications for national TB control programmes in ensuring that patients devotedly adhere to their drugs and come back for monthly follow-up. We also recommend that MDR-TB patients with treatment interruptions or lost to follow-up need to obtain mycobacterial culture results for  timely whole-genome sequencing. Our study also strongly highlights the importance of combating DR-TB in Africa through implementing next generation sequencing that can test resistance to all drugs while providing a faster turnaround time. This can facilitate timely clinical decisions in managing MDR-TB patients with non-adherence or lost to follow-up.

## Supplementary Information

**Additional file1.** Mycobacteria growth indicator tube (MGIT) drug susceptibility testing protocol.

**Additional file2.** Löwenstein-Jensen drug susceptibility testing protocol.

**Additional file3.** Detailed patient clinical and laboratory information.

## Data Availability

The raw reads and/or assembly files from this study are publicly available at the SRA under the study BioProject ID: PRJNA749651 and at the Zenodo open-access repository https://doi.org/10.5281/zenodo.5136879.

## Abbreviations

MTB: *Mycobacterium tuberculosis*
NTRL: National TB Reference Laboratory
WGS: Whole genome sequencing
TB: Tuberculosis
SRL: Supranational Reference Laboratory
XDR TB: Extensively drug resistant TB
