# Supplementary material for: First report of whole-genome analysis of an extensively drug-resistant Mycobacterium tuberculosis clinical isolate with bedaquiline, linezolid and clofazimine resistance from Uganda
Source: Antimicrob Resist Infect Control. 2022 May 12;11:68. doi: 10.1186/s13756-022-01101-2 (PMC9102340; doi:10.1186/s13756-022-01101-2)
Supplement: Supplementary file 1 — Additional file1. Mycobacteria growth indicator tube (MGIT) drug susceptibility testing protocol. [file 13756_2022_1101_MOESM1_ESM.pdf]

5. Principle of the analysis

Isolated cultures from TB patients are subjected to a known concentration of a test anti-TB drug. A growth control is also included with no addition of an anti-TB drug. This system uses the common proportion method, that is to say resistance is determined at a 1% level for the anti-TB drugs. Resistance is expressed as the percentage of growth index on drug containing media in comparison to the growth index on drug-free growth control. This means that 1% or more of the total test bacterial population is resistant to the test drug, it is considered as resistant for clinical purposes.

7.3 Reagents

- Sterile Distilled water
- Drugs (Citical concentration CC): Isoniazid (0.1 µg/ml), Rifampicin (0.5 µg/ml), and Ethambutol (5.0 µg/ml) (SIRE kit) & Pyrazinamide kit (100 µg/ml)
- Drugs (Critical concentration-CC and/or Critical Breakpoint-CB):
    - Routinely: Levofloxacin (LEV – 1.0 µg/ml), Moxifloxacin CC (MOX CC 0.25 µg/ml), Amikacin (AMK - 1.0 µg/ml), Bedaquilin (BDQ - 1.0 µg/ml), Linezolid (LZD-1.0 µg/ml) and Clofazimine (CFZ - 1.0 µg/ml)
    - Treatment failure at >Month 4 or On request: Delamanid (0.06 µg/ml), Moxifloxacin CB (high dose -1.0 µg/ml), Ethionamide 5.0 µg/ml, Prothionamide 2.5 µg/ml etc
- 0.4% NaOH

  NB: Preparation of the 0.4% NaOH

  To 2 mL of 6% NaOH, aseptically add 28 mL of SDW

  or

  To 2 mL of the 4% NaOH, aseptically add 18 mL of SDW

  Mix well. This is the 0.4% NaOH solution.
- DMSO
- BACTEC MGIT 960 SIRE Supplement
- BACTEC MGIT 960 tubes
- SD MPT-64 antigen test kit.

1.4 Quality controls

Run control strains H37Rv, MX(9990 ITM ROUND 24 2018), KA(4012 ITM ROUND 24 2018), BDQ (Round 25 ITM strain code 10), LZD (Round 25 ITM strain code 06) and DLD (Round 25 ITM strain code 07) when a new batch of drug working solution is prepared.

Strain	AMK	LEV	MOX	BDQ	LZD	CFZ	DLM	ETO	PTO	Other drug(s)
**H37RV**	S	S	S	S	S	S	S	S	S	S
**KA**	S	R	R	N/A	N/A	N/A	N/A	N/A	N/A	N/A

**MX**	R	R	R	N/A	N/A	N/A	N/A	N/A	N/A	N/A

Never set DST from a MGIT tube that has been positive for more than five days. It should be sub-cultured in a fresh MGIT tube supplemented with MGIT 960 growth supplement and should be tested in MGIT 960 instrument until it is positive. Use this tube from one to five days of instrument positivity for the subsquent MGIT DST process.

- To minimize environmental contaminants from being introduced during addition of growth supplement:
  a. Make sure all additions are made inside the biosafety cabinet.
  b. Do not open several tubes at a time.
  c. Open MGIT tube for as short a period of time as possible.
  d. Recap the tube tightly. If the cap is left loose, it may affect the detection of fluorescence.
  e. Do not add more than 500ul of specimen as this may disturb the pH of the medium and cause false fluorescence.
  f. Once the tubes are placed in the stations, they should not be twisted or turned.
  g. Tubes should not be removed except in the following conditions: removal of positives, removal of negatives and reassigning position if station becomes bad.

7.5 Work procedure

7.5.1 Reconstitution of BD lyophilized drugs

(i) How to constitute lyophilized drugs in MGIT. Follow table below

Drug	Needed conc (µg/ml)	Total µg in a drug/vial	Reconstitute vial with sterile distilled water	Resultant drug concetration (µg/ml)	Volume to be added in MGIT tube (ml)
Amikacin	1.0	332	4ml	83	0.1
Moxifloxacin	0.25	249	Dilute 1:4 i.e 1ml (of 1.0 µg/ml) into 3 ml of water	20.75	0.1<br>0.1
	1.0		3ml	83	0.1
Levofloxacin	1.0	249	3ml	83	0.1
Bedaquilin	1.0	170	2ml	83	0.1

7.5.3 Preparation of M. Tuberculosis growth inoculum

a. Inoculum from MGIT tube

NOTES

It is important that growth be fresh within the following recommended time frame.

a. The day the MGIT tube is positive by the instrument is considered **Day 0.**
b. The tube should be kept incubated for at least one more day (**Day 1**) before using for susceptibility testing (may be incubated in a separate incubator at 37ºC+-1ºC).

c. A positive tube may be used up to the fifth day (**Day 5**) after it becomes instrument positive.
d. If growth in a tube is of **Day 1** or **Day 2**, mix well to break up clumps (vortex). (Leave the tube undisturbed for about 5-10 minutes to allow for large clumps to settle to the bottom). Use the supernatant undiluted as the inoculum for the drug impregnated tubes.
e. If growth is **Day 3, 4, or 5**, (mix well to break up the clumps, let the large clumps settle for 5-10 minutes) and dilute 1.0 ml of positive broth with 4ml of sterile distilled water. This will be a 1:5 dilutions. Use this well-mixed, diluted culture mixture as the inoculum for the drug impregnated tubes.

In brief, the following are the guidelines:

Day 0-the MGIT tube is positive by the instrument. Re-incubate at least one more day.

Day 1 or 2-one or two days after instrument positive. Use undiluted for the impregnation into the drug containing tubes.

Day 3, 4 or 5-dilute 1:5 and use as innoculum for impregnation into the drug containing tubes.

Day 6 and on ward-Subculture in a fresh MGIT tube and follow the above guidelines.

b. Inoculum from growth on solid medium

It is important to have fresh growth on the solid medium, such as LJ slant (within 15 days of appearance of growth on the medium). Old cultures may affect the outcome of the DST test results.

I. <u>INDIRECT MGIT DST FROM LJ ISOLATES</u>

1. Use growth on solid medium which is not more than 15 days old (with 15 days of appearance of positive growth).
2. Aseptically, add some (8-10) sterile glass beads in a propelly labeled universal bottle.
3. Aseptically, add 3 ml of sterile distilled water saline.
4. Using a sterile plastic loops, scrape as many colonies as possible trying not to remove any of the solid medium.
5. Deposit the colonies into the univeral bottle by rubbing the loop against the insideside of the bottle such as to emulsify the colonies.
6. Tightly screw the cap
7. Vortex for 30 seconds
8. Using a sterile plastic Pasteur pipette, match the suspension with McFarland 0.5 Standard by adding sterile saline.
9. Inoculate a MGIT tube with 0.8 mL PANTA mixture added with 500 mL of the 0.5 McFarland match suspension
10. Load the MGIT tube into the MGIT machine and wait until it turns positive.
11. Perform ZN test and BA plate
12. If ZN positive perform rapid TB antigen test.
13. If ZN positive and no growth with BA plate and rapid TB antigen test positive proceed for MGIT DST.

II. <u>DIRECT MGIT DST FROM LJ ISOLATES</u>

1. Colonies from solid media may be used if they are no more than 15 days form the first appearance of positive growth.
2. Using a sterile plastic wire loop scrape as many colonies as possible trying not to remove any of the solid medium.
3. Deposit the colonies into the univeral bottle by rubbing the loop against the insideside of the bottle such as to emulsify the colonies.
4. Tighten the cap and vortex the tube for 2-3 minutes to break up any large clumps. The turbidity of the suspension should be greater than the McFarland number 1 standard.
5. Let the suspension stand for 20 minutes undisturbed.
6. Using a sterile pipette, carefully transfer the supernatant suspension into another sterile tube.
    a. Avoid taking any growth that has settled on the bottom.
7. Let this tube stand for another 15 minutes undisturbed.
8. Adjust the turbidity of this suspension to McFarland 0.5 by adding sterile distilled water and visually comparing with the 0.5 Mcfarland.
    a. The turbidity should not be less than McFarland 0.5
9. Dilute 1.0 ml of this suspension in 4.0 ml sterile distilled water and mix well.
10. This dilution is used as the inoculum for DST.

7.5.4 Procedure for preparation and inoculation of MGIT tubes

1. Depending on the type of AST carrier rack avaialbale or to be used i.e. 5 tube carrier or 8 carrier rack and number of second line drugs to be tested.
    a. If 8 tubes rack, all routine drugs (6) will be put in addition to a “dummy/plain tube” and QC.
    b. If 5 tube carrier rack: two racks will be required to run all the routine drugsd:
    NB: blank MGIT tubes (non-inoculated, drug-free) can be used to fi ll empty slots in a carrier.
2. Label MGIT-tubes for each of the test culture. Each of the drug tubes is labelled with the appropriate drug to be tested, e.g. label one for GC (growth control, without drug), one for AMK-Amikacin, LEV-Levofloxacin, MOX-Moxifloxacin, BDQ-Bedaquiline, LZ-Linezolid, Cfz-Clofazamine.
3. NB: the drugs set for DST may be interchaged with any of the availabe 2nd line drugs depending on the request/need of the physcian.
4. Aseptically add 800ul of BACTEC MGIT 960 SIRE Supplement to each of the MGIT tubes. **Use only MGIT SIRE Supplement and not MGIT Growth Supplement.**
5. Aseptically add 100ul of the appropropriate properly reconstituted drugs into the approprate tube. Use a separate micropipette tip for each drug. **Do not add any drug to the GC tube.**
6. Mix the sample by inversion of the tube 2-3 times.
7. Aseptically add 500ul of the inoculum, either the broth of the positive MGIT or its dilution 1:5 , depending on the number of days elapsed between the positivity and the test, into each of the tube containing the drugs,
8. Do not add to growthcontrol.
9. For the growth control, use a 1:100 dilution of the inoculum : add 100ul of either the broth of the positive MGIT or its dilution 1:5 (depending on the number of days passed between the positivity and the test) to 9.9 ml sterile distilled water.
10. Mix well by inverting the tube 2-3 times.
11. Add 500 ul of the 1:100 diluted suspension to the growth control tube.
    a. Tighten the tubes and then mix well by inverting the tube 2-3 times.

12. Place the MGIT-tubes in a 5-tube set carrier, paying special care in respecting the correct order, i.e GC tube is placed at the 1st slot of the carrier followed by the drug containing tubes.
13. Bring the set carriers to the Bactec MGIT 960 instrument for incubation.
14. Touch any of the membrane buttons on the screen to activate the screen
15. Open the desired drawer
16. Select the option **'tube entry'**
17. Scan the Set Carrier barcode; the dedicated positions are indicated on the screen and the green lights in the drawer are highlighted. Carefully enter the Set Carrier at the indicated positions. Make sure the tubes are completely inside the stations before closing the drawer.
18. Close the drawer and let the tubes be incubated for up to 13 days.

7.5.5 Remove completed MGIT DST tubes from the BACTEC-MGIT^{960} machine

1. The instrument monitors the entered susceptibility test set. Once the test is complete (within 4-13 days), the instrument will indicate that result as ready (it will light red-'positive').
2. Open the drawer that lights positive/red and then press the DST remove icon.
3. The tubes to be removed then light green. Remove the set carrier and scan the bar code. Repeat this step for all ready set DSTs.
4. Close the drawer.

7.6 Interpretation and validation of MGIT Results

The instrument print out will indicate susceptibility results for each drug. Results are qualitative as Susceptible (S), Resistant (R) or undetermined (X).

- The instrument interprets results at the time when GU in growth control reaches 400 (within 4-13 days). At this point, GU values of the drug vial are evaluated.
- **S=**Susceptible- GU of the drug tubes less than 100.
- **R=**Resistant-GU of the drug tube more than 100.
    - **X=**Error-undetermined results when certain conditions occur which may affect the test,-X ### = Error or Indeterminate results; reported when certain conditions occur that may affect the test. If possible, determine the cause of error. In any case, repeat testing with a pure culture of the isolate.
    - X200 = System cannot detect sufficient indication of growth in the Growth Control tube in the specified protocol time, and does not provide an interpretation of the AST set results. Often a result of too little inoculum, nonviable organisms, or a slow growing drug-resistant strain. Please refer to Flow Chart in Annex 2 for further instructions.
    - X400 = System detects indications of possible contaminated or over inoculated tube, and does not provide an interpretation of the AST set results. Check the tube for turbidity and subculture to a blood agar plate to rule out contamination of the specimen. Please refer to Flow Chart in Annex 3 for further instructions.
    - Other conditions, such as power failure.

Reasons for Repeat

MGIT 2nd line DST may be repeated for a number of reasons:

- Contamination or over innoculation of the GC or drug containing tubes (this will appear as X400 on the result print out)Under innoculations or slow growing strains of certain resistant bacteria that may not achieve results within 4-13 days with standard innoculum.

Trouble shooting

1. Observe all 'resistant' tubes visually for evidence of contamination when first removed from the instrument. Perform a ZN stain on any suspicious tube and subculture to a BAP. In addition, when drug resistance is observed and the patient's isolate has not been tested before, or if the isolate was not previously resistant to the drug, test tube(s) with ZN and BAP to ensure that growth is not due to contaminants or MOTT.Errors, e.g., x200 and x400, are generated the same as for first-line drugs depending upon the AST set configuration used for testing, and necessitate repeating following the flow charts in Annex 2 and 3.
2. DST for second line drugs should only be repeated once if the first test fails. If a valid result cannot be obtained after the second attempt, report the test as "TF", report results to the requestor with the appropriate comment in the LIS such as
   (i) Please provide another sample

   if DST fails on the two attempts available, DST should be done on the next available positive culture of the same patient.
3. If DST results for any of the fluoroquinolones or injectable agents are inconsistent with previous results for the same patient, review the results and QC. Use patient profile in the LIS to make a decision for repeat or not and repeat the test.
4. Where applicable, GeneXpert test maybe requested to rule out cross contamination of the original MGIT tube in cases where the sample is from a known Rifampicin resistant patient.
5. Use patient profile in the LIS to trouble shoot a result before release.

NB: Record results of ZN and BAP on the MGIT Machine DST print out against each lab ID number.

7.7 Subculture of isolates for MGIT second line DST

NB: Subcultures for repeat DST should always be inoculated on both LJ and MGIT culture to increases chances of recovery.

Subculture of original MGIT positive tube isolates for DST are done incases of:

- Contmination or under growth(X200 and X400) of the innoculated DST samples.
- Contaminated or old primary cultures beyound 5 days for MGIT culture and 14 days for LJ culture isolates. or the set DST.

Subculture of frozen crovial (from LJ or MGIT culture)

- Incase the original MGIT positive tube is compromised i.e. visible signs of contaminated, too old.
- Always remember to document on the isolate retrieval form.

7.8 Interferences

It is important that proper identification of Mycobacterium tuberculosis is carried out prior to setting the DST since Non Tuberculosis Mycobacteria-NTM can lead to false DST results (false resistant results).

7.9 Alert/Critical values

Extensively drug resistant-XDR TB results should immediately be reported to the Technical

Supervisor such that a thorough assessment and review of these results is made prior to reporting the results to the customer.

7.10 Turn around time

It takes 4-13 days for the DST results to be out once the assay has been initiated or once the tubes have been incubated in the MGIT machine.
