# Supplementary material for: First report of whole-genome analysis of an extensively drug-resistant Mycobacterium tuberculosis clinical isolate with bedaquiline, linezolid and clofazimine resistance from Uganda
Source: Antimicrob Resist Infect Control. 2022 May 12;11:68. doi: 10.1186/s13756-022-01101-2 (PMC9102340; doi:10.1186/s13756-022-01101-2)
Supplement: Supplementary file 2 — Additional file2. Löwenstein-Jensen drug susceptibility testing protocol. [file 13756_2022_1101_MOESM2_ESM.pdf]

Uganda National Tuberculosis and Leprosy Program

National Tuberculosis Reference Laboratory
LJ DST protocol

1. **Drug Susceptibility Testing (DST) by Modified Proportional Method on Löwenstein-Jensen Media**
   - **Abbreviations, definitions and terms**
   - AM/A Amikacin
   - BSC II Bio Safety Cabinet class II
   - DST Drug Susceptibility Testing
   - EMB/E Ethambutol
   - INH/H/I Isoniazid
   - ITM Institute of Tropical Medicine
   - LJ Löwenstein-Jensen
   - LEV/L Levofloxacin
   - LPA Line Probe Assay
   - MDR-TB Multi Drug Resistant-Tuberculosis
   - MOTT Mycobacteria Other than Tuberculosis
   - N.A. Not applicable
   - NTLP National Tuberculosis and Leprosy Control Programme
   - NTM Non Tuberculosis Mycobacterium
   - NTRL National Tuberculosis Reference Laboratory
   - MOX/M Moxifloxacin
   - PNB Para Nitro Benzoic acid
   - RMP/R/Rif Rifampicin
   - XDR-TB Extensively Drug Resistant-Tuberculosis

2. **Definitions**

MDR: Multi-Drug Resistant Tuberculosis (MDR-TB)
Defined as TB that has developed resistance to at least both of the following first-line anti TB drugs: Rifampicin and Isoniazid.

PRE-XDR: TB caused by *Mycobacterium tuberculosis (M. tuberculosis)* strains that fulfil the definition of multidrug resistant and rifampicin-resistant TB (MDR/RR-TB) and which are also resistant to any fluoroquinolone.

XDR: Extensively Drug Resistant Tuberculosis (XDR-TB)
Defined as TB caused by *Mycobacterium tuberculosis (M. tuberculosis)* strains that fulfil the definition of MDR/RR-TB and which are also resistant to any fluoroquinolone (Levofloxacin or moxifloxacin) and at least one additional Group A drug (Group A drugs are the most potent group of drugs in the ranking of second-line medicines for the treatment of drug-resistant forms of TB using longer treatment regimens and comprise levofloxacin, moxifloxacin, bedaquiline and linezolid).

3. **Principle of the analysis**

The method aims on determining the proportion of resistant bacilli present in a strain. The number of colonies growing on drug free medium is compared to the number on drug containing medium and the proportion of resistance bacilli are determined.

4. **Procedure**

Indication for DST
1. Presumptive MDR cases
   - Rif resistance by GeneXpert or LPA
2. As requested by clinician

3. MDR-TB treatment follow-ups with positive cultures month 4 and above

5.1 Sample analysis

Colonial appearance identified as belonging to *Mycobacteria Tuberculosis* Complex, growing on Löwenstein-Jensen medium, **not older than 2 weeks** from LJ culture reading date are the ones suitable for LJ DST. All LJ isolates that are indicated for LJ DST and are either contaminated or too old should first be sub-cultured on LJ media and recorded in the sub culture register before setting on LJ DST.

5.2 Equipment

1. Biosafety cabinet
2. Calibrated sterile plastic Pasteur pipette (3ml and 1ml)
3. Biohazard bags
4. Sterile 14 ml bijou bottles
5. Sterile Khan tubes
6. Vortex
7. Glass beads
8. Incubator
9. Sterile plastic loops
10. Refregirator
11. N95 respirators

5.3 Reagents/Isolates/Control strains

1. Media LJ containing Rifampicin 40µg/ml properly labeled (drug, specimen number) which has passed the QC.
2. Media LJ containing Ethambutol 2µg/ml properly labeled (drug, specimen number) which has passed the QC
3. Media LJ containing Isoniazid 0.2µg/ml and 1µg/ml respectively properly labeled drug, drug concentration, specimen number which has passed the QC.
4. Media LJ containing Para Nitro Benzoic acid (500 µg/ml) properly labeled (drug, specimen number) which has passed the QC.
5. Media LJ free of drug properly labeled (date, concentration of the inoculum to be added, specimen number) which has passed the QC.
6. If required, for second line drug testing, media LJ containing Levofloxacin 2.0µg/ml properly labeled (date, concentration of the inoculum to be added, specimen number) which has passed the QC.
7. If required, for second line drug testing, media LJ containing Amikacin 30 µg/ml properly labeled (date, concentration of the inoculum to be added, specimen number) which has passed the QC.
8. If required, for second line drug testing, media LJ containing Moxifloxacin 1.0 µg/ml properly labeled (date, concentration of the inoculum to be added, specimen number) which has passed the QC.
9. No. 1 McFarland standard
10. Bacterial dilutions of *Mycobateria fortutituim,* , H37RV, MX and KA), culture/test isolates

5. Quality control

1. Anti TB drug QC table

Control slopes must show the below drug pattern for the test to pass.

Strain	INH	RIF	ETH	LEV	AM	MOX	PNB
1. H37Rv	S	S	S	S	S	S	No growth
2. MX	S	S	S	R	R	R	No growth
3. KA	R	R	R	R	S	R	No growth

NB:

(i) Drug patterns depends on the control strains and PT strains obtained from ITM-Belgium in March 2013 and PT round 24 2018

Note:

- <1+ (Less than 1+ growth) means a range from no growth to 1+ growth.
- Control strains H37Rv cater for sensitivity of both first line and second line drugs.
- Control strains MX and KA caters for sensitivity and resistance on both first and second line drugs

2. PNB pattern QC table

Strain	Plain LJ	PNB
Mycobacterium fortuitum	Growth	Growth
Mycobacterium TB complex	Growth	No growth QC-PNB

3. The growth of the plain LJ growth control, inoculated with 10^{-2} suspension should have a colony count between 2+ to 3+. If less or more, the results are non interpretable (inoculum too weak or too strong, that may lead to false susceptible or false resistance results respectively)
4. The growth of the plain LJ growth control, inoculated with 10^{-4} suspension should have a colony count between 5 and 100 colonies. If less or more, the results are non interpretable.

6. DST Setting:

1. Retrieve positive isolates from the incubator which are indicated for LJ DST on form A004 F3 (Samples that require DST)
2. Prepare bacteria suspensions of the LJ isolates to be inoculated using 1.0 McFarland to standardize the suspensions (Refer to SOP P038 (Preparation and dillution of Bacterial Suspensions).
3. When setting first line drugs and second line drugs, arrange the different types of slopes in n in the diagram below

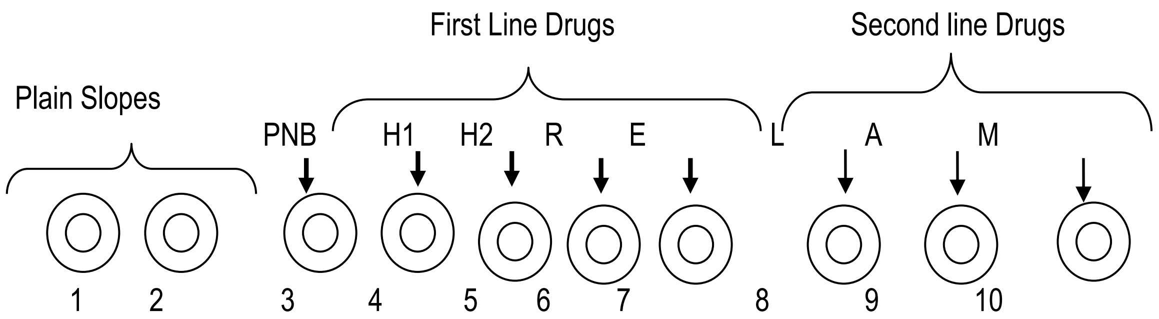

4. Label slopes with reference strain or test isolate Laboratory number and place them in the tray as in the table 1, below.

Table 1

	Control 1	Control 2	PNB	H1	H2	R	E	L	A	M
Dilution / Isolate	10^{-2}	10^{-4}	10^{-2}	10^{-2}	10^{-2}	10^{-2}	10^{-2}	10^{-2}	10^{-2}	10^{-2}
M.fortutium			59							
H37Rv										
MX										
KA										
Test isolate 1										
Test isolate 2										

Note: Second line drugs are set along side with First line drugs depending on the request

Ensure that the plain LJ media and DST media batches have passed QC before. DST QC can be set along with the first batch of routine samples.

Work in a certified BSCII. For the safety measures, refer to SOP 12, safety Manual.

5. Prepare dilutions using pasteur pipettes of 3ml afor transferring 2.25ml volume) and 1 ml, (for transferring 0.25ml volume) to sterile glass khan/test tubes or any other that may be available. (Refer to SOP P038, Preparation and dillution of Bacterial Suspensions).

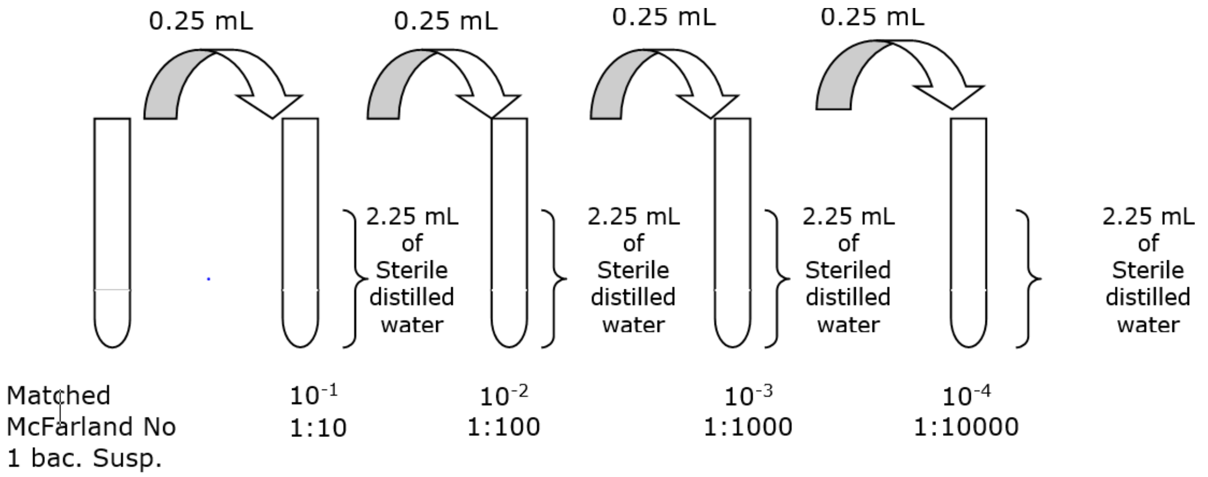

6. Before Inoculating on any of the slopes, aseptically pour off all the water of condensation from the side of the universal bottle without the media.
7. Inoculate the slopes with 100 µl of the corresponding bacterial dilutions (Refer to the table 1 above) using a Pasteur pipette as follows;

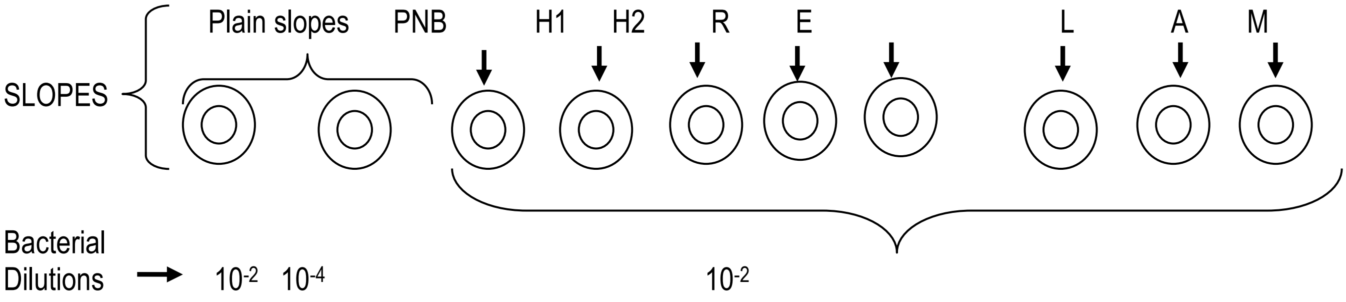

- After inoculation complete LJ DST setting and result worksheet and Form A004 F1(LJ DST setting and results worksheet) and Form A004 F2 (LJ DST setting and results worksheet continuation)
- Transfer the inoculated slopes into the incubator.
- Incubate at 37°C for 4 - 6 weeks, read final results for PNB and Ethambutol at 4 weeks and the rest for 4 – 6 for the rest of the drugs.
  - The tech in the section should check out for contamination of the incubated LJ DST cultures on a weekly basis.The tech should retrieve the isolate for a contaminated sample, subculture and record in the LJ subculture register .

8. **DST Culture Reading, interpreting and reporting**

8.1 Reading Scale: (according to the International Union Against Tuberculosis and Lung Diseases IUATLD)

**Reading (colonies)**	***Report***
Contaminated	*Contaminated*
1 – 50	*Actual number*
51 -100	*1+*

More than 1+, but still numerable (101 – 299)	2+
300-500 (innumerable colonies)	3+
>500 (confluent growth)	4+

Before reading the tests, one must confirm that the batch used has passed QC (see section Quality Control)

Record the results on the LJ DST setting and result worksheet, Form A004 F1&2 (LJ DST Setting and results worksheet)

8.2 Examining the cultures

1. The results can be read in two steps. The results are read 28 days (4 weeks) and 42 days (6 weeks) after inoculation.

- Count the total number of colonies growing on the different tubes. *Control 1* usually has confluent growth; *Control 2* should have countable colonies (about 20-100 colonies). If two tubes have been inoculated as controls then an average of the colony count for the two is used for calculations.
- The proportion of resistant bacilli is calculated by comparing the counts on Control 1 (with the 10^{-2} dilution) with the counts on the medium containing the anti-TB agent (with the 10^{-2} dilution). Then, a percentage is calculated using the following formula.

$$\frac{\text{Number of colonies on the medium containing the anti-TB agent}}{\text{Number of colonies on Control 1 } (10^{-2} \text{ dilution})} \times 100$$

Note: *Do not count colonies that are growing only on the upper part of the slant because this indicates that the anti-TB agent has been inactivated in that portion.* ***The number of colonies grown on Control 1 must be approximately 100 times the number of colonies on Control 2; this indicates that the 1:100 dilution has been prepared properly.***

The growth of the plain LJ growth control, inoculated with 10^{-2} suspension should have a colony count between 2+ to 3+. If less or more, the results are non interpretable (inoculum too weak or too strong, that may lead to false susceptible or false resistance results respectively)

2. Control 1 (10^{-2}) usually has confluent growth; Control 2 (10^{-4}) should have countable colonies (about 20-100 colonies).
3. Only plain LJ controls inoculated with 10^{-2} and 10^{-4} suspensions should be used for interpretation of the resistance and sensistivity patterns on the drug slants.
4. **4.** The bench reviewer should always compare the realtime result with the patient profile before the final result is reported.

8.3 Interpreting Results

1. PNB test

Any growth with at least 1+ grade is interpreted as positive, regardless of the 10^{-4} control. Any positive results indicate that the strain used is entirely or partially containing non tuberculosis mycobacteria. The DST results obtained for this particular strain must be rejected.

Because antigen and molecular identification tests will be positive in presence of mixed organisms (MTB complex and MOTT/NTM), PNB is recommended for all LJ DST.

2. Other drugs

Results are interpreted using the 1% proportion method. This means that if 1% or more of the populations are resistant then the culture is reported as resistant. If the ratio of the number of colonies on the medium containing an anti-TB agent (at the critical concentrations of the drugs to the number of colonies on the control medium is less than 1%, then the strain is reported as susceptible.

- If on day 28 the ratio of the number of colonies on the medium containing the anti-TB agent to the number of colonies on the control medium is 1% or greater, then the culture is resistant. If there are no colonies on the medium containing the anti-TB agent and Control 1 (10^{-2}) has confluent growth, the strain can be reported as ***SUSCEPTIBLE*** without further reading. Except for these two circumstances, all other results should be reported after day 40.
- On day 28 if Control 1 (10^{-2}) or Control 2 (10^{-4}) has 20 colonies or fewer, this indicates that the medium has been insufficiently inoculated. If there is growth on the medium containing the anti-TB agent it can be reported as ***RESISTANT***, but if there is no growth on the medium containing the anti-TB agent, the test should be ***REPEATED*** using fresh inoculum.
- If Control 2 (10^{-4}) has confluent growth, this indicates that the medium has been overinoculated, and the test should be ***REPEATED***. In this situation, growth on the medium containing the anti-TB agent could be due to over inoculation, and the culture should not be interpreted as ***RESISTANT***. However, if there is no growth on the medium containing the anti-TB agent, the test can be reported as ***SUSCEPTIBLE***.
- Results of the tests should be reported only as ***"SUSCEPTIBLE" OR "RESISTANT"*** to facilitate use by the clinician. Results should be reported as soon as they are available. Results can be reported after 4 weeks if there is growth on the medium containing the anti-TB agent and all other parameters are satisfactory.

9. Reason to repeat test

- QC Failure: Technical supervisor calls for an emergency meeting with section team and other technical experienced staff to brainstorm the possible causes, narrow down to a possible root cause and interventions, then fill the occurence management form.
- For any LJ DST result that fails QC, the outcome must be filled in the LJ DST Setting and Result Worksheet and OMF filed.
- Repeating of samples: Isolates with inconclusive drug interpretation are repeated on LJ DST to come up with conclusive sensitivity or resistance patterns.
- Occurence management form is written and client notified about the delay or asked to provide another sample if possible.

10. Performance specifications

95% Efficicency for rifampicin and isoniazid and 90% efficiency for Ethambutol and 90% for second line drugs (Moxifloxacin, Levofloxacin and Amikacin).

11. Turnaround time

4-6 weeks after culture positive.
