# Supplementary material for: First report of whole-genome analysis of an extensively drug-resistant Mycobacterium tuberculosis clinical isolate with bedaquiline, linezolid and clofazimine resistance from Uganda
Source: Antimicrob Resist Infect Control. 2022 May 12;11:68. doi: 10.1186/s13756-022-01101-2 (PMC9102340; doi:10.1186/s13756-022-01101-2)
Supplement: Supplementary file 3 — Additional file3. Detailed patient clinical and laboratory information. [file 13756_2022_1101_MOESM3_ESM.docx]

**SUPPLEMENTARY material three**

| **Lab ID:** | 84873 | 85671 | 86501 | **Registration #1 narrative**  Patient came in as F1, tested RR on 30/07/2017, CT value for GeneXpert was not indicated in the issued Lab report from the lower facility.  The patient had a Negative HIV serostatus, Chest X-Ray showed heterogeneous opacities bilaterally, with obliterated costophrenic angles bilaterally.  Before initiation on DR-TB treatment on 31-July-2017, a sputum sample was collected and sent to the National TB Reference Laboratory for Culture and Drug Susceptibility Testing (DST).  Initial Regimen: 6Km Lfx Cs Eto Z /14 Lfx Cs Eto Z Baseline weight: 50Kgs   \| ***DRUG*** \| ***Z*** \| ***Km*** \| ***Cs*** \| ***Lfx*** \| ***Eto*** \| \| --- \| --- \| --- \| --- \| --- \| --- \| \| ***DOSE*** \| ***1200mg*** \| ***800cc*** \| ***500mg*** \| ***1000mg*** \| ***750mg*** \|   Declared LTFU on: 12-December-2017  Justification for lost to follow up: He was last reviewed at the monthly MDR-TB clinic at LRRH MDR-TB ward, on the 25/10/2017, his known mobile phone numbers went off, no one knew his whereabouts till he came back in May 2018 |
| --- | --- | --- | --- | --- | --- | --- | --- | --- | --- | --- | --- | --- | --- | --- | --- | --- |
| **Sample Collected** | 31-Aug-2017 | 27-Sep-2017 | 25-Oct-2017 |  |
| **Month** | M0 | M1 | M2 |  |
| **Microscopy** | 3+ AFBs Seen | Negative | Negative |  |
| **LJ culture** | 3+ Colonies | 1+ Colonies | NA |  |
| **MGIT culture (TTD)** | Positive (4day: 12hours) | Positive(19days:4hours) | Negative |  |
| **LPA 1 FLQ** | Sensitive٭ | NA | NA |  |
| **LPA 2 AG/CP** | Sensitive٭ | NA | NA |  |
| **Phenotypic DST** | **MGIT DST 1^st^ Line**  Streptomycin-Sensitive  Isoniazid-Resistant  Rifampicin-Resistant  Ethambutol-Sensitive |  |  |  |
| **Phenotypic DST** | **MGIT DST 2^nd^ Line**  Amikacin-Sensitive  Kanamycin-Sensitive  Moxifloxacin-Sensitive  Capreomycin-Sensitive |  |  |  |

**Table 1:** Patient profile for the monthly sputum smear, culture and Drug Susceptibility Testing conducted from 31st August 2017 to 25th October 2017.

| **Lab ID:** | 91739 | 92458 | 93383 | 94121 | 95105 | 96032 | 97055 | 98534 |
| --- | --- | --- | --- | --- | --- | --- | --- | --- |
| **Sample Collected** | 11-May-2018 | 06-Jun-2018 | 11-Jul-2018 | 1-Aug-2018 | 08-Sep-2018 | 03-Oct-2018 | 07-Nov-2018 | 10-Jan-2019 |
| **Month (Date)** | **M0** | M1 | M2 | M3 | M4 | M5 | **M6** | M8 |
| **Microscopy** | 3+ AFBs Seen | Negative | Negative | Negative | Negative | Negative | Negative | Negative |
| **LJ culture** | 3+ Colonies | 7 Colonies | Negative | Negative | Negative | Negative | 2+ Colonies | 3+ Colonies |
| **MGIT culture (TTD)** | Positive (5days:7hours) | Positive(12days:5hrs) | Positive(18days:22hours) | Negative | Negative | Negative | Positive (10days:11hours) | Positive |
| **LPA 1 FLQ** | Sensitive٭ | NA | NA | NA | NA | NA | Resistant٭٭ | NA |
| **LPA 2 AG/CP** | Sensitive٭ | NA | NA | NA | NA | NA | Sensitive٭ | NA |
| **Phenotypic DST** | **LJ DST 1^st^ Line**  Streptomycin-Sensitive  Isoniazid-Resistant  Rifampicin-Resistant  Ethambutol-Sensitive |  |  |  |  |  | **MGIT DST 1^st^ Line**  Streptomycin-Resistant  Isoniazid-Resistant  Rifampicin-Resistant  Ethambutol-Sensitive |  |
| **Phenotypic DST** | **LJ DST 2^nd^ Line**  Kanamycin/Ofloxacin/  Capreomycin/Amikacin- Sensitive |  |  |  |  |  | **MGIT DST 2^nd^ Line**  Amikacin-Sensitive  Kanamycin-Sensitive  Levofloxacin-Resistant  Moxifloxacin-Resistant |  |

| **Lab ID:** | 99185 | 100136 | 100946 | 101848 | 102677 | 103663 | **Registration #2 narrative**  Patient was re-registered on: 12/05/2018  Started on new regimen that includes:  *6Km Lfx Cs Eto Z /14 Lfx Cs Eto Z*  Culture converted at: **M4**(08-09-2018)  Then reverted at: **M6**(07-11-2018)  Declared failure on: 3/6/2019  DST reversion: Resistance to Streptomycin and Fluoroquinolones.  Noted: Patient had serious adherence issues (Very poor adherence) |
| --- | --- | --- | --- | --- | --- | --- | --- |
| **Sample Collected** | 06-Feb-2019 | 06-Mar-2019 | 03-Apr-2019 | 08-May-2019 | 05-Jun-2019 | 03-Jul-2019 |  |
| **Month (Date)** | M9 | M10 | M11 | **M12** | M13 | M14 |  |
| **Microscopy** | Negative | Neg | 3+ AFBs Seen | Neg | Negative | Negative |  |
| **LJ culture** | Negative | Neg | 3+ Colonies | 3+ Colonies | 2+ Colonies | Negative |  |
| **MGIT culture (TTD)** | Negative | Neg | Positive | Positive (8days:19hours) | Positive (6days:23hours) | Positive (26days:11hours) |  |
| **LPA 1 FLQ** | NA | NA | NA | Resistant٭٭ | NA | NA |  |
| **LPA 2 AG/CP** | NA | NA | NA | Sensitive٭ | NA | NA |  |
| **Phenotypic DST** |  |  |  | **MGIT DST 1^st^ & 2^nd^ Line**  Streptomycin-Resistant  Isoniazid-Resistant  Rifampicin-Resistant  Ethambutol-Sensitive  Amikacin-Sensitive  Levofloxacin-Resistant  Moxifloxacin-Resistant |  |  |  |
| **Phenotypic DST** |  |  |  | **MGIT DST (New Drugs)**  Bedaquiline-Sensitive  Clofazimine-Sensitive |  |  |  |

**Table 2:** Patient profile for the monthly sputum smear, culture and Drug Susceptibility Testing conducted from 11-May-2018 to 03-July-2019.

| **Lab ID:** | 104795 | 106109 | 108920 | 109793 | 110533 | 111648 | 111960 | **Registration #3 narrative**  Patient was re-registered on: 29/7/2019  National Panel recommended: 24 Bdq Dlm Lzd Eto Cs Z  Had culture conversion at: **M5**  Then reverted at: **M6**  Declared failure on: 6/05/2020  DST reversion: Resistance to Streptomycin and Fluoroquinolones.  Declared LTFU: **after M10**  No monthly smear and culture follow-up samples collected at Month 3, 4, and 8. |
| --- | --- | --- | --- | --- | --- | --- | --- | --- |
| **Sample Collected** | 07-Aug-2019 | 25-Sep-2019 | 18-Dec-2019 | 29-Jan-2020 | 26-Feb-2020 | 29-Apr-2020 | 27-May-2020 |  |
| **Month (Date)** | M1 | M2 | M5 | M6 | M7 | M9 | M10 |  |
| **Microscopy** | 1+ AFBs Seen | Negative | Neg | Negative | Negative | Negative | Negative |  |
| **LJ culture** | 3+ Colonies | Negative | Neg | Negative | 3 Colonies | Negative | Negative |  |
| **MGIT culture (TTD)** | Positive (12days:0hours) | Negative | Neg | Positive (20days:1hour) | Positive  (14days:3hours) | Negative | Negative |  |
| **LPA 1 FLQ** | Resistant٭٭ | NA | NA | NA | NA | NA | NA |  |
| **LPA 2 AG/CP** | Sensitive٭ | NA | NA | NA | NA | NA | NA |  |
| **Phenotypic DST** |  |  |  |  | **LJ and MGIT DST 1^st^ Line**  Isoniazid-Resistant  Rifampicin-Resistant  Ethambutol-Sensitive |  |  |  |
| **Phenotypic DST** |  |  |  |  | **MGIT DST 2 + Newer drugs**  Amikacin-Sensitive  Levofloxacin-Resistant  Moxifloxacin-Resistant  Bedaquiline-Sensitive  Clofazimine-Sensitive  Linezolid-Sensitive |  |  |  |

**Table 3:** Patient profile for the monthly sputum smear, culture and Drug Susceptibility Testing conducted from 07-Aug-2019 to 27-May-2020

| **Lab ID:** | 112923 | 113447 | 113968 | 114997 | 115536 | **Registration #4 narrative**  Patient was re-registered on: 2/6/2020  New regimen: 12 Bdq Lzd Dlm Cfz Cs /6 Lzd Cfz Cs  Showed Resistance to Bedaquiline, Clofazimine and Linezolid at M2-3  Declared a LTFU on: 2/2/2021 and is nowhere to be seen up to date.  No monthly smear and culture follow-up sample collected at month 4 |
| --- | --- | --- | --- | --- | --- | --- |
| **Sample Collected** | 22-Jul-2020 | 26-Aug-2020 | 23-Sep-2020 | 11-Nov-2020 | 17-Dec-2020 |  |
| **Month (Date)** | M1 | M2 | M3 | M5 | M6 |  |
| **Microscopy** | Negative | Negative | Negative | Negative | Negative |  |
| **LJ culture** | 1+ Colonies | 1+ Colonies | 1+ Colonies | Negative | Negative |  |
| **MGIT culture (TTD)** | Negative | Positive (15days:23hours) | Positive(21ays:14hours) | Negative | Contaminated |  |
| **LPA 1 FLQ** | Resistant٭٭ | NA | NA | NA | NA |  |
| **LPA 2 AG/CP** | Sensitive٭ | NA | NA | NA | NA |  |
| **Phenotypic DST** | **LJ DST 1**  Isoniazid-Resistant  Rifampicin-Resistant  Ethambutol-Resistant | **MGT DST 1^st^ Line**  Pyrazinamide-Sensitive | **MGIT DST 1^st^ Line**  Isoniazid-Resistant  Rifampicin-Resistant  Ethambutol-Sensitive |  |  |  |
| **Phenotypic DST** | **LJ DST 2**  Amikacin-Sensitive  Levofloxacin- Resistant  Moxifloxacin- Resistant | **MGIT DST 2^nd^ Line**  Amikacin-Sensitive  Levofloxacin-Resistant  Moxifloxacin (CB)-Resistant  Bedaquiline-Resistant  Clofazimine-Resistant  Linezolid- Resistant | **MGIT DST 2^nd^ Line**  Amikacin-Sensitive  Levofloxacin-Resistant  Moxifloxacin-Resistant  Bedaquiline-Resistant  Clofazimine-Resistant  Linezolid- Resistant |  |  |  |

**Table 4:** Patient profile for the monthly sputum smear, culture and Drug Susceptibility Testing conducted from 22-Jul-2020 to 17-Dec-2020

**For LPA Results Interpretation:**

٭ Sensitive = Resistance Not Detected

٭٭Resistance = Resistance Detected

**KEY:**

AFBs = Acid Fast Bacilli

M6- Month and number

CB = Critical Breakpoint Concentration applies to high-dose Moxifloxacin

AG/CP = Injectable antibiotics (Kanamycin, Amikacin/Capreomycin, Viomycin)

FLQ = Fluoroquinolones (Ofloxacin, Moxifloxacin)

LJ = Lowenstein-Jensen

MGIT = Mycobacterium Growth Index Tube

TTD = Time to Detection

DST = Drug Susceptibility Testing

| **Year** | **2017** | **2018** | | **2019** | **2020** | | |
| --- | --- | --- | --- | --- | --- | --- | --- |
| **Date Sample Collected** | 31-Aug-2017 | 11-May-2018 | 07-Nov-2018 | 08-May-2019 | 26-Feb-2020 | 26-Aug-2020 | 23-Sep-2020 |
| **Month (Date)** | M0 | **M0** | **M6** | **M12** | **M7** | M2 | M3 |
| **Microscopy** | 3+ AFBs Seen | 3+ AFBs Seen | Negative | Negative | Negative | Negative | Negative |
| **LJ culture** | 3+ Colonies | 3+ Colonies | 2+ Colonies | 3+ Colonies | 3 Colonies | 1+ Colonies | 1+ Colonies |
| **MGIT culture (TTD)** | Positive (4day: 12hours) | Positive (5days:7hours) | Positive (10days:11hours) | Positive (8days:19hours) | Positive(14days:3hours) | Positive (15days:23hours) | Positive (21days:14hours) |
| **LPA 1 FLQ** | Sensitive٭ | Sensitive٭ | Resistant٭٭ | Resistant** | NA | NA | NA |
| **LPA 2 AG/CP** | Sensitive٭ | Sensitive٭ | Sensitive٭ | Sensitive٭ | NA | NA | NA |
| **Phenotypic DST** | **MGIT DST 1^st^ Line**  Streptomycin-Sensitive  Isoniazid-Resistant  Rifampicin-Resistant  Ethambutol-Sensitive | **LJ DST 1^st^ Line**  Streptomycin-Sensitive  Isoniazid-Resistant  Rifampicin-Resistant  Ethambutol-Sensitive | **MGIT DST 1^st^ Line**  Streptomycin-Resistant  Isoniazid-Resistant  Rifampicin-Resistant  Ethambutol-Sensitive | **MGIT DST 1^st^ Line**  Streptomycin-Resistant  Isoniazid-Resistant  Rifampicin-Resistant  Ethambutol-Sensitive | **LJ and MGIT DST 1^st^ Line**  Isoniazid-Resistant  Rifampicin-Resistant  Ethambutol-Sensitive | **MGIT DST 1^st^ Line**  Pyrazinamide-Sensitive | **MGIT DST 1^st^ Line**  Isoniazid-Resistant  Rifampicin-Resistant  Ethambutol-Sensitive |
| **Phenotypic DST** | **MGIT DST 2^nd^ Line**  Amikacin-Sensitive  Kanamycin-Sensitive  Moxifloxacin-Sensitive  Capreomycin-Sensitive | **LJ DST 2^nd^ Line**  Kanamycin/Ofloxacin/  Capreomycin/Amikacin- Sensitive | **MGIT DST 2^nd^ Line**  Amikacin-Sensitive  Kanamycin-Sensitive  Levofloxacin-Resistant  Moxifloxacin-Resistant | **MGIT DST 2^nd^ Line**  Amikacin-Sensitive  Kanamycin-Sensitive  Levofloxacin-Resistant  Moxifloxacin-Resistant  Bedaquiline-Sensitive  Clofazimine-Sensitive  Linezolid-Sensitive | **MGIT DST 2 + Newer drugs**  Amikacin-Sensitive  Kanamycin-Sensitive  Levofloxacin-Resistant  Moxifloxacin-Resistant  Bedaquiline-Sensitive  Clofazimine-Sensitive  Linezolid-Sensitive | **MGIT DST 2^nd^ Line**  Amikacin-Sensitive  Kanamycin-Sensitive  Levofloxacin-Resistant  Moxifloxacin (CB)-Resistant  Bedaquiline-Resistant  Clofazimine-Resistant  Linezolid- Resistant | **MGIT DST 2^nd^ Line**  Amikacin-Sensitive  Kanamycin-Sensitive  Levofloxacin-Resistant  Moxifloxacin-Resistant  Bedaquiline- Resistant  Clofazimine-Resistant  Linezolid-Resistant |
| **Regimen** | **6Km Lfx Cs Eto Z /14 Lfx Cs Eto Z** | **6Km Lfx Cs Eto Z /14 Lfx Cs Eto Z** | | **24 Bdq Dlm Lzd Eto Cs Z** | | **12 Bdq Lzd Dlm Cfz Cs /6 Lzd Cfz Cs** | |
|  |  |  |  |  |  | **Samples selected for Sequencing** | |

Table 5: Patient TB positivity timelines for the monthly sputum smear, culture and Drug Susceptibility Testing conducted between Aug 2017 to Sep 2020
